# A NiCo-MOF nanosheet array based electrocatalyst for the oxygen evolution reaction†

**DOI:** 10.1039/d0na00112k

**Published:** 2020-04-01

**Authors:** Ponmuthuselvi Thangasamy, Saravanakumar Shanmuganathan, Viswanathan Subramanian

**Affiliations:** Department of Industrial Chemistry, School of Chemical Sciences, Alagappa University Karaikudi-630003 Tamil Nadu India rsviswa@gmail.com

## Abstract

Metal organic frameworks (MOFs) are excellent materials for energy storage and conversion. This report describes 2D metal–organic framework nanosheets as an electrocatalyst for the oxygen evolution reaction (OER) under alkaline conditions. An ultrathin nanosheet array of a NiCo-metal–organic framework was grown on nickel foam (NiCo-MOF/NF) by a one-step solvothermal method. The catalytic OER of the NiCO-MOF/NF electrode was analysed by electrochemical methods. The resulting NiCO-MOF/NF exhibited a high current density (50 mA cm^−2^) with an overpotential of 270 mV, a Tafel slope of 35.4 mV dec^−1^ and a high turnover frequency (TOF) of 0.68 s^−1^ (*η* = 0.27 V) towards the OER. The excellent catalytic activity of the MOF towards the OER was due to the two-dimensional nanosheet array of NiCo-MOF with plentiful accessible molecular active sites and excellent mass transport properties. Faster electron transport was also achieved due to the synergetic effect of Co and Ni present on the MOF.

## Abstract 2

Metal organic frameworks (MOFs) are excellent materials for energy storage and conversion.

## Graphical abstract

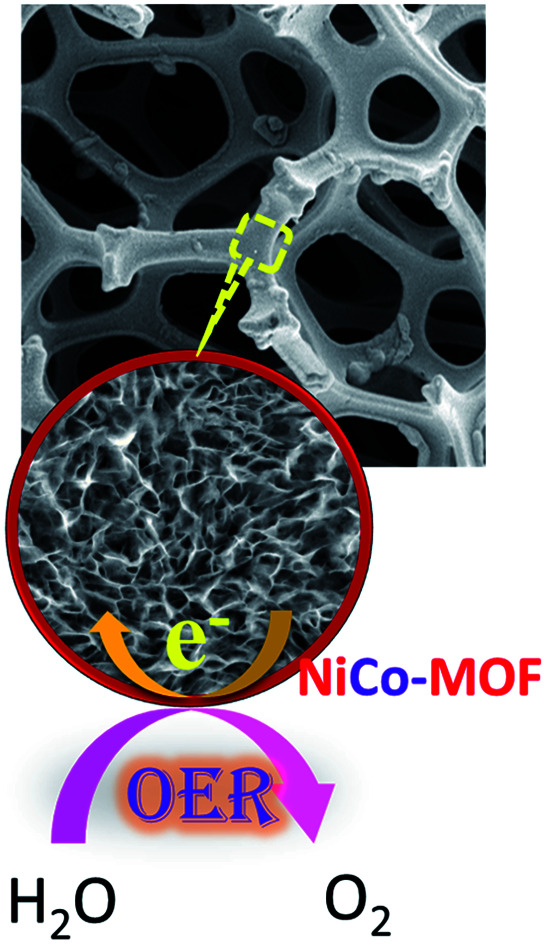

## Introduction

Metal–organic frameworks (MOFs) are crystalline porous materials constructed by the coordination of metal ions or clusters and organic linkers. In recent years, MOFs received tremendous attention in gas storage applications and active catalytic materials for energy-related applications due to their tunable porosity and readily accessible active sites.^1^ Recent literature findings suggested that calcination at a high temperature may sacrifice MOF-derived electrocatalysts that possess intrinsic metal active sites, while hybridization with secondary supports, such as graphene, polyaniline and carbonaceous materials, may block their intrinsic micropores.^2^ In addition, bulk MOFs have a limited micro and mesoporosity for an effective mass transport during electrocatalytic activity. 2D MOF nanosheets possess numerous accessible active sites, meso/macroporosity and a faster electron transfer, which are essential criteria to design high-performance electrocatalysts.^3^ The merits of 2D-nanomaterials, such as plentiful active centres to improve the high catalytic activity and large surface area to volume ratio enable rapid mass transport and charge transfer.^4^ Moreover, the strategy focusing on the water splitting reaction using MOFs as the electrode materials supported on secondary phases, such as self-supported materials on graphene,^5^ black phosphorous,^6^ Ni-foam^7^ and Mxenes,^8^*etc.*, is rapidly growing. Energy conversion and storage technologies (water splitting, metal–air batteries, and fuel cells) are totally dependent on highly active electrocatalysts.^9–11^ However, inherently very sluggish reaction kinetics have been observed due to the four-electron transfer steps and a large overpotential is required for the reaction. Precious noble metal-based electrocatalysts, such as RuO_2_ and IrO_2_, are the benchmark catalysts for OER, but they hardly satisfy scale-up applications because of their scarcity and high cost.^3^ Therefore, there is a critical need for cost-effective alternatives with efficient OER catalysts based on earth-abundant metals, such as transition metal oxides,^12^ hydroxides^13^ and carbonaceous materials.^14^ Among these, nickel-based OER catalysts, including hydroxides,^15^ sulfides^16^ and phosphides,^17^ have demonstrated better OER catalytic performances under alkaline conditions. In particular, bimetallic cobalt and Ni-based compounds display enhanced catalytic performances.^18,19^ Recent investigation suggests metal–organic frameworks are highly suitable for OER applications. 2D nanosheet based electrodes received wide attention due to their nanostructure with a high catalytic activity^20,21^ and emerged as excellent alternatives for OER applications. NiFe based MOF nanosheets directly supported on nickel foam acting as robust electrodes for an electrochemical oxygen evolution reaction was reported by Sun *et al.*^21^ Duan *et al.* demonstrated that the electrocatalyst based on bimetallic MOF nanosheets have highly exposed active molecular metal sites with an enhanced catalytic performance for water splitting reactions.^7^ Herein, we describe a simple method for the *in situ* growth of an ultrathin two-dimensional Ni–Co-MOF nanosheet array on nickel foam (NiCo-MOF/NF). The NiCo-MOF/NF electrode shows an OER with a small overpotential of 270 mV at a current density 50 mA cm^−2^ and is stable for 30 000 seconds without any noticeable activity decay.

## Results and discussion

**Fig. 1:**
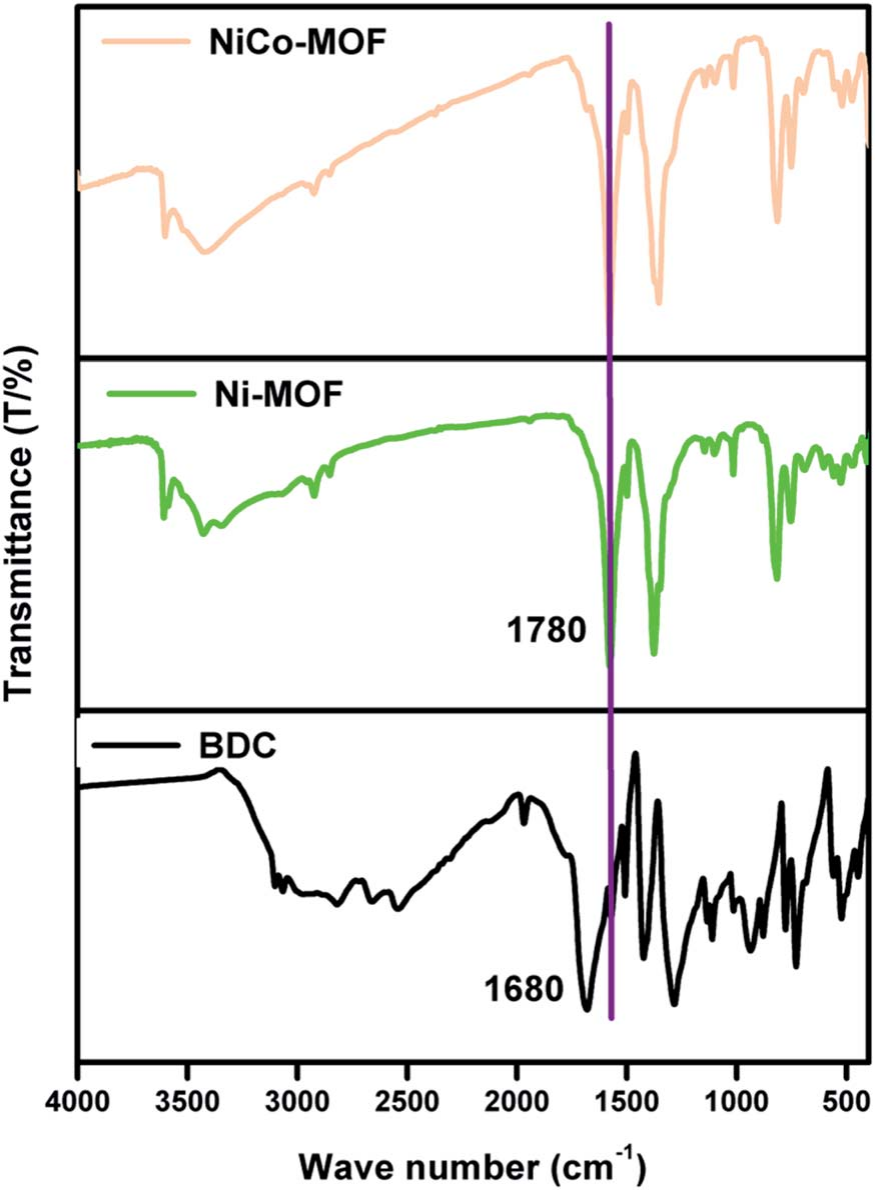
FT-IR spectra of NiCo-MOF, Ni-MOF and the BDC ligand.

FT-IR analyses were performed to investigate the participation of functional groups in the formation of MOFs. Fig. 1 shows two peaks at 1680 and 1424 cm^−1^ which are characteristic peaks of the ligand H_2_BDC (due to the asymmetric and symmetric (–COO^−^) stretching vibrations). The peaks at 1680 and 1424 cm^−1^ are absent in the case of Ni-MOF and NiCo-MOF, thereby confirming that the ligands are coordinated with a metal centre. Further, NiCo-MOF (Fig. 1) shows peaks at 1579 and 1354 cm^−1^, corresponding to the asymmetric and symmetric (–COO^−^) stretching vibrations of the ligand coordinated to the metal centre. The peaks at 3424 and 1499 cm^−1^ are ascribed to the stretching vibration of OH^−^ and *para*-aromatic CH groups, respectively. The appearance of peaks (Ni–O and Co–O) at 474, 603 and 699 cm^−1^ corresponds to the formation of the metal–oxo bond between the (Ni or Co) atoms and the carboxylic group of the H_2_BDC ligand. The above results reveal the formation of a metal centre (Ni^2+^, Co^2+^) coordinated with an organic linker (BDC^2−^). The FT-IR spectra of Ni-MOF and Co-MOF are shown in ESI Fig. 1.†

**Fig. 2:**
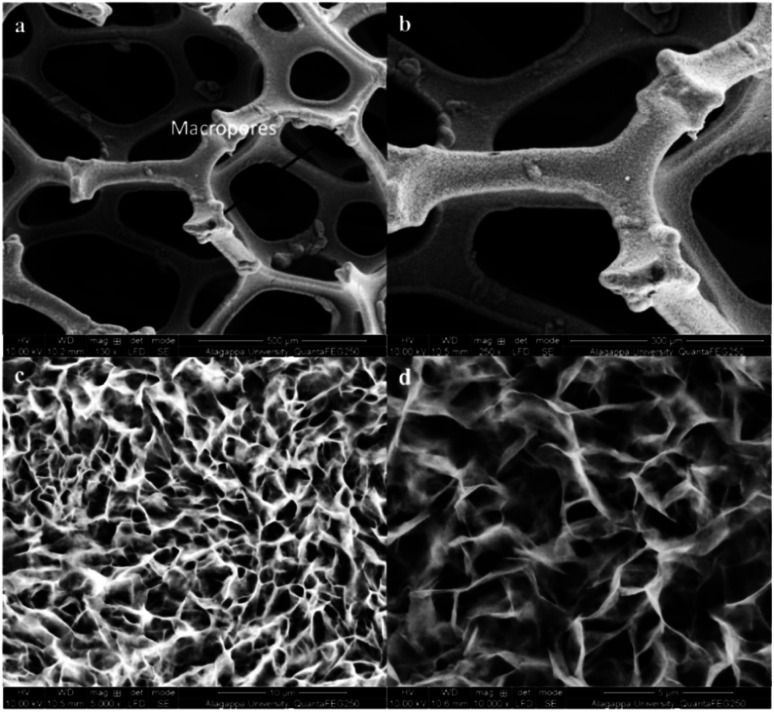
FE-SEM images of NiCo-MOF/NF at various scales: (a) 500 μm, (b) 300 μm, (c) 10 μm, and (d) 5 μm.

The Ni-foam substrate is highly flexible and mechanically robust with numerous macropores with the pore sizes of 200–500 μm. The surface morphology of NiCo-MOF was characterized by field emission scanning electron microscopy (FE-SEM). The FE-SEM images of NiCo-MOF (Fig. 2a and b) confirmed the formation of ultrathin nanosheets uniformly arranged on Ni-foam. Ni-MOF/NF was synthesised in a similar solvothermal process except for CoCl_2_·6H_2_O. The figure (ESI Fig. 2†) displays the density of a 3D-parallelogram slice of Ni-MOF growing on Ni-foam, which obviously differs from that of 2D-NiCO-MOF/NF.

**Fig. 3:**
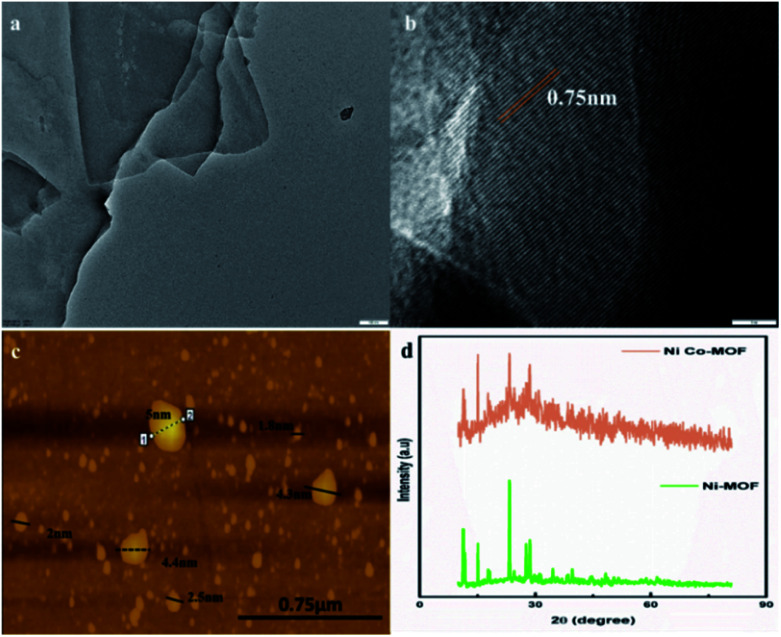
(a) and (b) HR-TEM images (scale bars: 100 nm and 5 nm, respectively), (c) an AFM image, and (d) the P-XRD pattern of the NiCo-MOF nanosheet array.

HR-TEM images of NiCo-MOF indicate the 2D nanosheet array formation with a well-defined ultrathin sheet-like structure (Fig. 3a). The 2D MOF nanosheets exhibit a crystalline structure, as evidenced by the clear lattice fringes (0.75 nm lattice space, Fig. 3b). The 2D-NiCoMOF nanosheet array formation was confirmed by AFM. The thickness of the NiCo-MOF sheet was found to be ∼2–5 nm (Fig. 3c) with only a few molecular nanolayers stacked.

XRD of Ni-MOF and Ni–Co-MOF nanosheets was carried out and the results are depicted in the Fig. 3. The XRD pattern of Ni-MOF revealed peaks at 2*θ* values of 11.29°, 11.72°, 15.06°, 23.29°, 28.58°, and 34.51°. These peaks are well-matched with crystalline data CCDC: 638866.^22^ The XRD pattern of the NiCo-MOF exhibited peaks at 2*θ* values of 15°, 23.7°, 28.9° and 51° that belong to the (111), (220), (311) and (511) planes, respectively. The diffraction results for NiCo-MOF were verified with the reported JCPDS no: 73-1704 data.^23^ A similar pattern was obtained for bimetallic NiCo-MOF. Co ion insertion altered the triclinic crystal structure of Ni-MOF to a face-centred cubic crystal structure. These results confirmed that the nickel and cobalt ions are involved in the MOF formation.

**Fig. 4:**
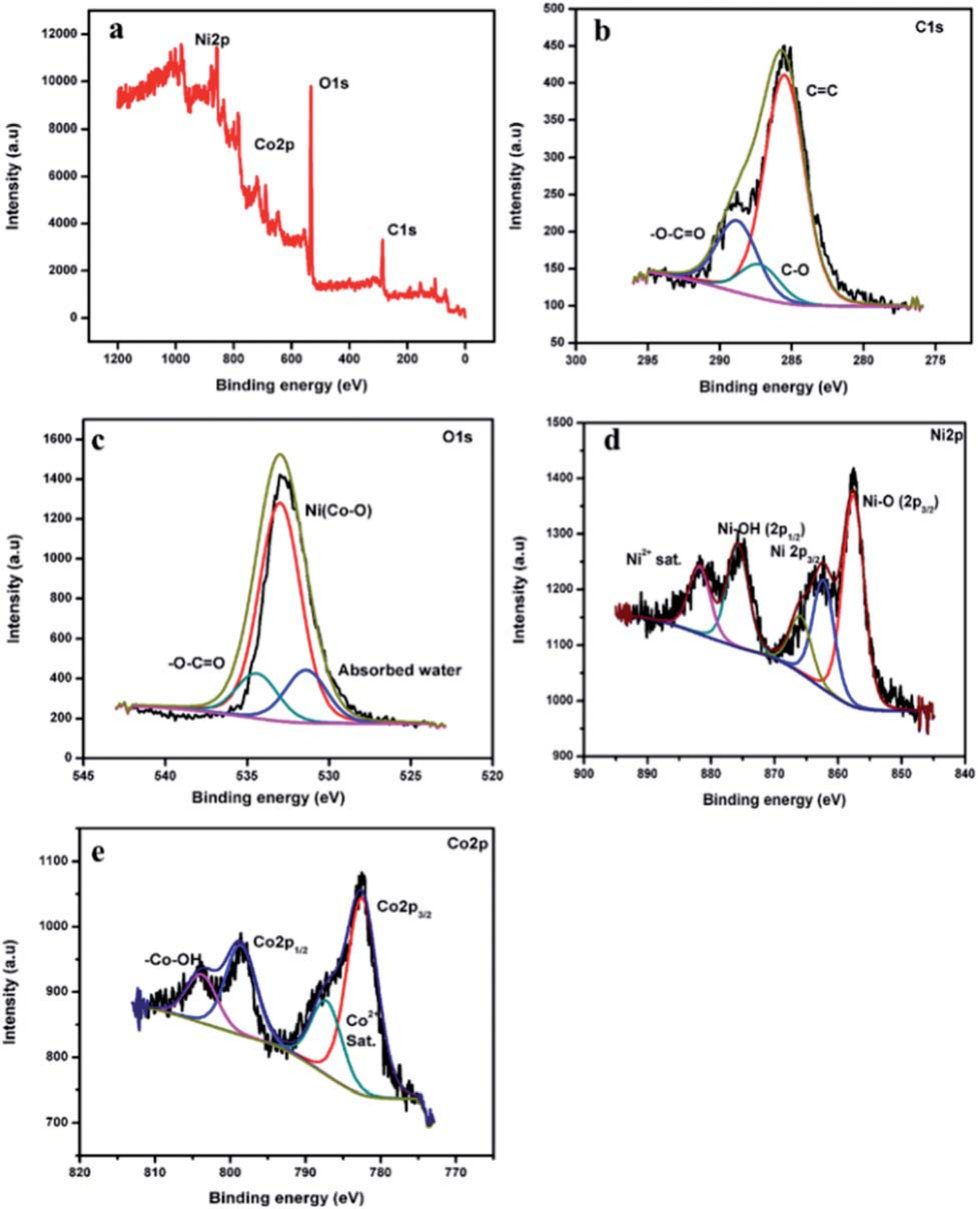
(a) XPS survey spectra of NiCo-MOF/NF, and (b–e) C 1s, O 1s, Ni 2p and Co 2p high resolution spectra, respectively.

X-ray photoelectron spectroscopy (XPS) analysis was carried out to find the surface composition and the surface electronic states for NiCo-MOF and investigate the charge transfer processes in the valence state of the nickel and cobalt ions of NiCo-MOF, as well as synergetic effect of nickel and cobalt species (Fig. 4a).

The XPS spectrum of NiCo-MOF/NF shows the presence of C, O, Ni and Co elements. The high-resolution XPS (HR-XPS) spectrum of C 1s is deconvoluted into the three surface components corresponding to the benzene rings of the BDC ligand (285.6 eV), the carboxylate rings of the (O=C–O–) groups of the organic ligand (288.8 eV), and –C–O (287.2 eV).^21^Fig. 4c is the XPS spectrum of O 1s fitted by two peaks at binding energies 532.9 and 534.3 eV, which can be attributed to the presence of oxygen atoms on the Ni and Co–O bonds and the carboxylate moiety of the organic ligand, respectively. HR-XPS of Ni 2p and Co 2p spectra both show 2p_1/2_ and 2p_3/2_ components due to spin–orbit coupling. The high-resolution Ni 2p spectra of the NiCo-MOF/NF show characteristic peaks of the Ni^2+^ oxidation state. The binding energy of 857.4 eV (2p_3/2_) was assigned to the Ni–O corresponding to the MOF structure, while the binding energy peak around 875.5 eV (2p_1/2_) was assigned to Ni–OH.

The binding energy peaks around 862.6 (2p_3/2_) and 882.4 eV were ascribed to the satellite peak of Ni 2p. The high-resolution Co 2p spectrum of NiCo-MOF/NF (Fig. 4e) displays two peaks corresponding to Co 2p_3/2_ and Co 2p_1/2_ at 782.0 eV and 798.3 eV, respectively. The binding energy values of 804.4 eV and 787.9 eV were assigned to –Co–OH and the satellite peak of Co 2p. From the XPS data (Fig. 4a), the chemical composition of the NiCo-MOF material exhibited that carbon (43.78 at%), oxygen (45.42 at%), nickel (6.60 at%) and cobalt (4.19 at%) are present.

**Fig. 5:**
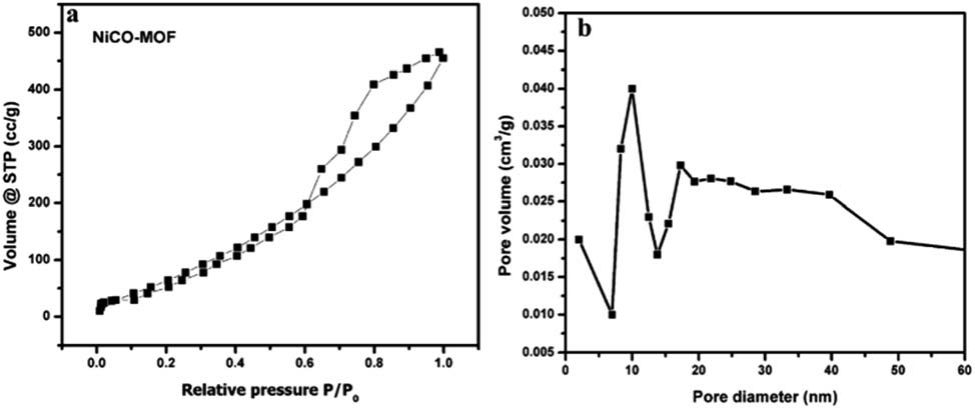
(a) N_2_ adsorption–desorption isotherms of the NiCo-MOF nanosheet array, and (b) the corresponding pore size determination of the NiCo-MOF nanosheet array.

Fig. 5 shows that NiCo-MOF nanosheets have type IV nitrogen adsorption/desorption isotherms, which indicates that the nanosheets are rich in mesoporous. The prepared 2D-nanosheets showed the higher surface area of 380 m^2^ g^−1^ and the Brunauer–Emmett–Teller (BET) surface area shows mesoporosity and the average diameter of pore size is 12 nm. The high surface area and abundant mesoporous nature of NiCo-MOF nanosheets provide benefits for the access of the electrolyte with OER active sites.

**Fig. 6:**
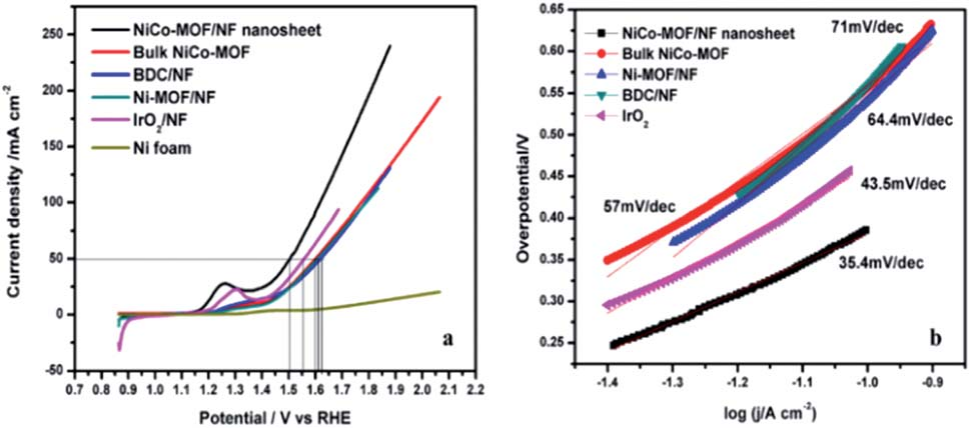
(a) Polarization curves and (b) the corresponding Tafel slopes of NiCo-MOF/NF, Ni-MOF, bulk NiCo-MOF and the BDC ligand.

The electrocatalytic performance of the NiCo-MOF/NF nanosheet array towards the oxygen evolution reaction was investigated using a standard three-electrode cell containing 1.0 M KOH. In prior OER experiments, all the prepared samples were subjected to cyclic voltammetry (10 cycles) at a sweep rate of 50 mV s^−1^ to initiate the pre-activation process. For comparison, electrochemical studies of the Ni-MOF/NF, Co-MOF/NF, BDC/NF, and Ni/CO-MOF/NF nanosheets, of Ni-MOF, Co-MOF, and Ni/Co-MOF synthesized by an *in situ* route and coated on the Ni-foam and of the benchmark catalyst IrO_2_ on Ni foam were performed under the same conditions. Fig. 6a shows the linear sweep voltammetric results (LSV) of the as-prepared above-mentioned catalysts. The Ni/Co-MOF/NF nanosheet array exhibited an overpotential of 270 mV at a current density 50 mA cm^−2^ which is extremely smaller than that of Ni-MOF/NF (380 mV), BDC/NF (395 mV), Ni/CO-MOF coated on the Ni-foam (370 mV, bulk), calcined NiCo-MOF (at 400 C for 4 h in N_2_ gas, 454 mV) and IrO_2_ (320 mV). The low overpotential of the NiCo-MOF/NF nanosheet array confirms its excellent OER activity due to the results from the flexible electron transfer across the fine sheet arrangement with sheets that are vertically arranged on the nickel foam.

Proposed mechanisms for the NiCo-MOF catalysed OER activity:

1 L(Co)NiOOH + OH^−^ ↔ L(Co)NiO(OH)_2_ + e^−^

2 L(Co)NiO(OH)_2_ + 2OH^−^ ↔ L(Co)NiOO_2_ + 2H_2_O + 2e^−^

3 L(Co)NiOO_2_ + OH^−^ → L(Co)NiOOH + O_2_ + e^−^

4 Overall: 4OH^−^ ↔ 2H_2_O + O_2_ + 4e^−^

Steps (1) and (2) may involve reversible process. Step (3) is fast, irreversible and also the rate determining step of the entire process. Presumably, NiCo-MOF is faster because of the reaction kinetics relating to step (3). In the event of anodic OER, LCo(NiO_4_) inside MOF was oxidised into L(Co)NiOOH species which promotes the oxidation of OH^−^ into O_2_.^9^ The outstanding electrocatalytic activity of the NiCo-MOF nanosheet is also evidenced through the turnover frequency (TOF) of 0.68 s^−1^, with the current density at a low overpotential of 270 mV, which is twice that of Ni-MOF (0.35 s^−1^, with the current density at a low overpotential of 380 mV) and IrO_2_/NF (0.58 s^−1^, with the current density at an overpotential of 320 mV).

The NiCo-MOF/NF nanosheet array exhibited a better catalytic activity than that of other electrodes towards OER with a Tafel slope of 35.4 mV dec^−1^ (Fig. 6). This slope value is much lower than those of other compositions samples, such as NiCo-MOF bulk (57 mV dec^−1^), Ni-MOF/NF (64.4 mV dec^−1^), BDC/NF (71 mV dec^−1^) and IrO_2_ (43.5 mV dec^−1^). These results confirm that the NiCo-MOF/NF nanosheet array exhibited an excellent catalytic activity compared to other existing catalysts owing to the intrinsically highly active sites and hierarchical porous structure, which permit a high surface area and easy to transport gaseous products.

**Fig. 7:**
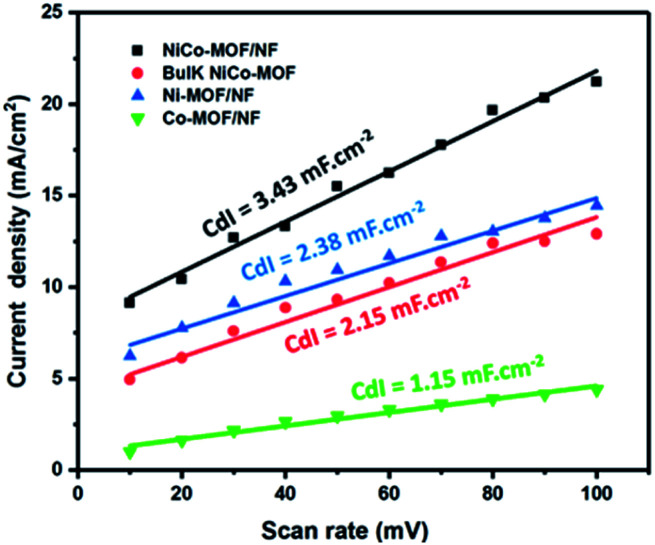
Electrochemically active surface area (ECSA) evaluation.

Another possible reason for the enhanced electrocatalytic activity after the addition of Co, the electrochemically active surface area (ECSA), was evaluated and is proportional to the double layer capacitances (*C*_dl_). Different scan rates of cyclic voltammograms were performed in the potential range of 0.9 to 1.2 V (*vs.* the reversible hydrogen electrode (RHE)) without a redox process in various electrodes, such as NiCo-MOF/NF, Ni-MOF/NF, Co-MOF/NF and bulk NiCo-MOF. The *C*_dl_ was calculated as *i* = *νC*_dl_, where *i* is the double layer current measured by cyclic voltammograms at different scan rates from 10 mV to 100 mV.^24^ From the results, the best electrocatalytic OER activity of NiCo-MOF/NF could be related to a major ECSA, which is beneficial for the contact area of the electrode and electrolyte (Fig. 7).

**Fig. 8:**
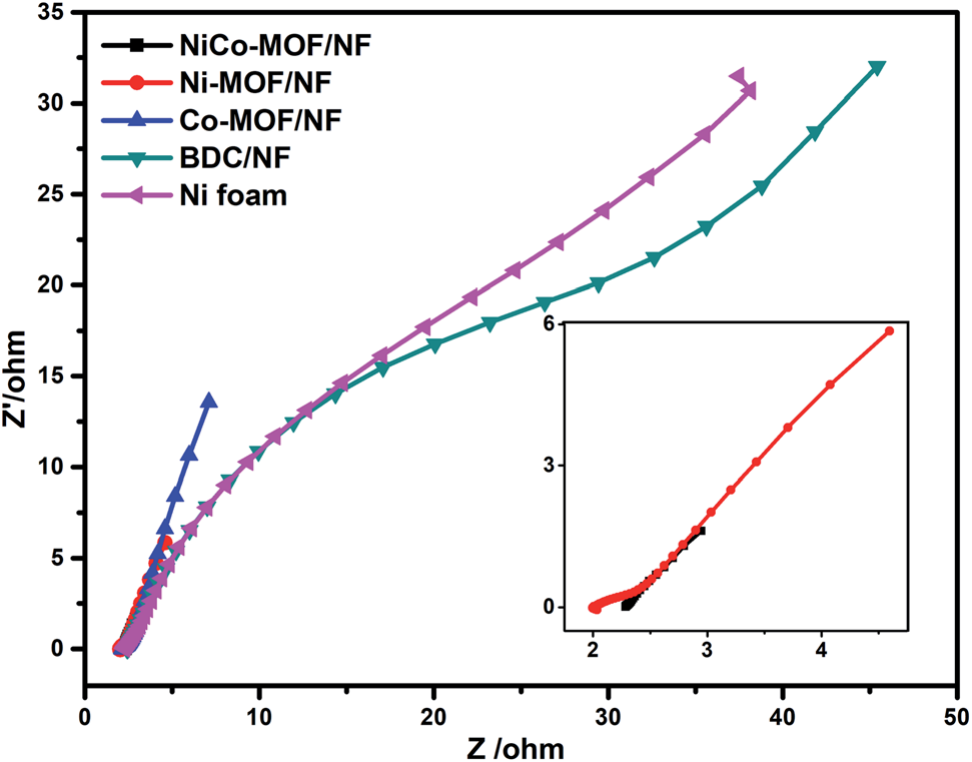
EIS results at 1.5 V *versus* RHE for NiCo-MOF/NF, Ni-MOF, bulk NiCo-MOF, BDC/NF.

Electrochemical impedance spectroscopy (EIS) consists of two frequency regions: a semicircle at a higher frequency and linear at low frequencies. The semicircle corresponds to faradaic resistance and is caused by interfacial charge transport in the electrode interface. EIS of the NiCO-MOF/NF, Ni-MOF/NF, Co-MOF/NF, BDC/NF, and Ni-foam were measured in a solution of 1 M KOH. As shown in Fig. 8, the semicircle diameter of the NiCo-MOF/NF nanosheet array is much smaller than those of other catalysts such as Ni-MOF/NF, Co-MOF/NF, BDC/NF, and Ni-foam, which demonstrates that the NiCo-MOF/NF electrode has a smaller electronic resistance other than the other electrodes. These results support that the incorporation of Co atoms into the Ni-MOF/NF structure can enhance the electron transport properties of the electrode–electrolyte interface, which enhances the electrocatalytic process.

Literature results were compared with those for NiCo-MOF/NF developed in this work and are listed in Table 1. The NiCo-MOF/NF nanosheet array shows a lower over potential, lower Tafel slope and higher stability than those reported in the literature.

**Table 1:** A comparison of the OER activity of the 2D NiCo-MOF/NF nanosheet array and recently reported electrocatalysts

Sample	*E*_onset_ potential (V)	Overpotential	Tafel slope (mV dec^−1^)	Substrate	Electrolyte	Reference
2D NiCo-MOF/NF nanosheet	1.39	270 mV @ 50 mA cm^−2^	35.4	Ni-foam	1 M KOH	This work
Bulk NiCo-MOF	1.44	370 mV @ 50 mA cm^−2^	57	Ni-foam	1 M KOH	This work
IrO_2_	1.42	320 mV @ 50 mA cm^−2^	43.5	Ni-foam	1 M KOH	This work
2D Co-MOF nanosheet	1.41	263 mV at 10 mA cm^−2^	74	GCE	1 M KOH	25
2D Ni-MOF@Fe-MOF nanosheet	—	275 mV at 10 mA cm^−2^	54	GCE	1 M KOH	20
Ultrathin CoMn-LDH	1.5	350 mV @ 10 mA cm^−2^	43	GCE	1 M KOH	26
Ultrathin Co(OH)_2_ (D-U-Co(OH)_2_) nanoarrays	—	228 mV @ 10 mA cm^−2^	57	Ni-foam	1 M KOH	27
CoNi (20 : 1)-P-NS@NF	—	273 mV @ 10 mA cm^−2^	52	Ni-foam	1 M KOH	28
Co–Ni–Se/C on Ni foam	—	300 mV @ 50 mA cm^−2^	63	Ni-foam	1 M KOH	29
Ni^II^Fe^III^@NC	—	360 mV @ 10 mA cm^−2^	—	Graphite carbon surface	0.1 M KOH	30
NiCoLDH/CP	1.53	367 mV @ 10 mA cm^−2^	40	Carbon paper	1 M KOH	31
Ni_*x*_Co_3−*x*_O_4_ nanowire array/Ti foil	—	370 mV @ 10 mA cm^−2^	120	Ti foil	1 M NaOH	32

The major highlight of this work is that the NiCo-MOF nanosheets (∼2–5 nm Fig. 3) possess highly active sites on their surface which could be enhanced by catalytic activity. NiCo-MOF was directly grown on Ni-foam, which is a combination of macropores, mesoporous and intrinsic microporosity. The macropores of nickel foam facilitated the mass transport of electrolytes and dissipated the gaseous products. The mesopores of the NiCo-MOF nanosheet array provide plentiful accessible active sites and easy ion diffusion pathways (inward OH^−^ ion and outward O_2_ bubbles).

XPS analysis investigated the charge transfer processes in the valence state of nickel and cobalt ions of NiCo-MOF. XPS confirmed that the valence electronic configuration of Co^2+^ is 3d^7^ with a high spin state and therefore Co^2+^ has unpaired electrons in a pi-symmetry (t_2g_). The d-orbital of Co^2+^ interacts with the bridging O through π-donation. Ni^2+^ ions have more electrons in π-symmetry (t_2g_) d-orbitals relative to those in Co^2+^. This property will increase the electron-donating ability of the π-symmetry lone pairs of the bridging oxygen atoms by repulsion. After hybridization, the charge is transferred from cobalt to nickel through organic ligands. These lattices offer a more stable environment for metal ions. A supplementary reason explains the increase in the Ni^2+/3+^ redox potential when Co^2+^ ions are incorporated into Ni-MOF (ESI Fig. 5†). After the incorporation of cobalt ions in the MOF structure they enhance the OER performance due to the synergistic coupling between Ni and Co. Finally, the binder-free NiCo-MOF/NF nanosheet array electrode minimises the resistance of the catalyst and overpotential due to the interaction of the catalysts and conductive NF.

### Stability and durability

**Fig. 9:**
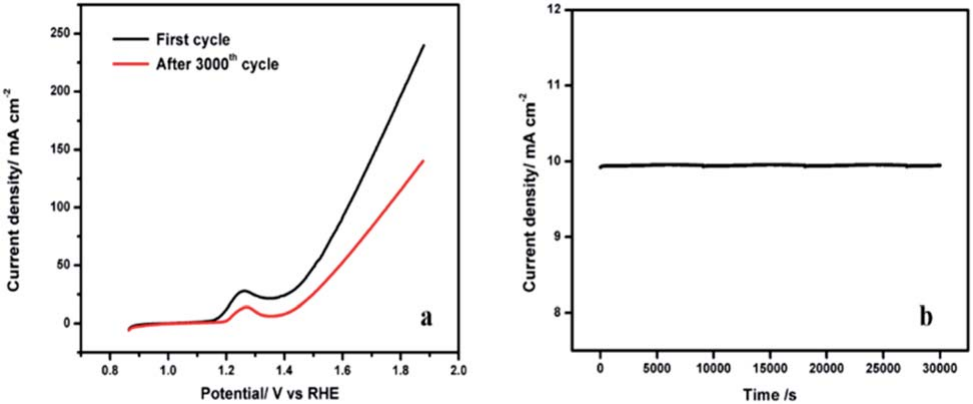
(a) Polarization curves of NiCo-MOF/NF before and after 3000 cycles in 1.0 M KOH. (b) The chronoamperometric curve of NiCo-MOF/NF at 1.5 V.

The stability of the NiCo-MOF/NF nanosheet array was evaluated by continuous CV for 3000 cycles at a scan rate of 50 mV s^−1^. After 3000 cycles, no significant change (*η* = 50 mV increase at a current density of 50 mA cm^−2^) in the current density was noticed at the NiCo-MOF/NF nanosheet array electrode (Fig. 9a). The long-term durability of the NiCo-MOF/NF nanosheet array was also tested by the chronoamperometric test at 1.5 V for 30 000 s (Fig. 9b). These results indicated that the developed electrode is stable and suitable for OER. Additionally, the morphology and crystal structure of the NiCo-MOF nanosheets are completely maintained after the chronoamperometric measurements (ESI Fig. 3 and 4†), proving the excellent stability.

## Conclusions

In summary, this work demonstrates a simple one step strategy to synthesise a 2D NiCo-MOF nanosheet array on the surface of Ni-foam. The structural morphology was examined *via* FT-IR, PXRD, FE-SEM, HR-TEM, BET, and XPS. The electrocatalytic properties of the NiCo-MOF/NF nanosheet array towards the OER under alkaline conditions were tested. The NiCo-MOF/NF nanosheet array electrode exhibited a higher OER performance, with a current density of 50 mA cm^−2^, a low overpotential of 270 mV and a Tafel slope of 35.4 mV dec^−1^. The OER electrocatalytic activity observed in this work is due to the high accessible surface area and the synergetic effect between Co and Ni. In addition, excellent electrode stability is observed due to the *in situ* growth of an ultrathin film MOF on the nickel foam. These results revealed that the MOF nanosheet array is an excellent electrocatalyst for the OER under alkaline conditions.

## Experimental details

### Synthesis of the NiCo-MOF/NF electrode

**Scheme 1:**
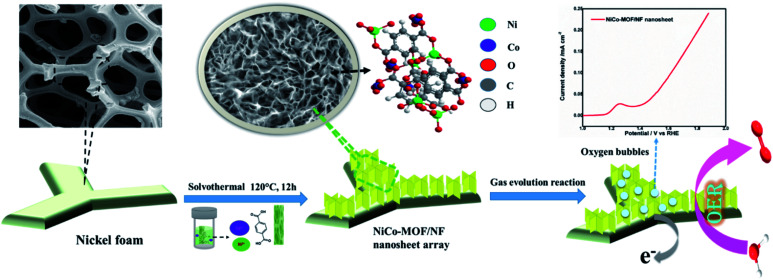
A schematic illustration of the synthesis of the NiCo-MOF/NF nanosheet array.

The bimetallic Ni–Co-MOF material was synthesised by mixing solutions of Ni^2+^, Co^2+^ salts together with benzene 1,4-dicarboxylic acid (BDC) *via* a one-step solvothermal method. Briefly, a piece of the nickel foam (1 cm × 1 cm) was washed successively with dilute HCl solution and acetone in an ultrasonic bath for 20 minutes respectively to remove the surface oxide layer and organic residue. After cleaning with Millipore water, it was then dried at 60 °C for 30 minutes. Nickel chloride (NiCl_2_·H_2_O, 7 mg), cobaltous chloride (CoCl_2_, 3 mg) and 1,4-benzene dicarboxylic acid (BDC, 10 mg) were dissolved in a 10 mL mixture solution of DMF/ethanol/water (5 : 3 : 2). After continuous ultrasonication for 30 minutes to obtain a clear solution, the mixture solution was transferred into a 25 mL Teflon-lined stainless steel vessel and the dried NF was placed in an autoclave. Then, the autoclave was sealed and maintained at 120 °C for 12 h in an oven. After attaining room temperature, the nickel foam was taken out from the Teflon container and ultra-sonicated for 5 min and then rinsed with deionised (DI) water to get the 2D NiCO-MOF nanosheet array (Scheme 1).

### Synthesis of Ni-MOF/NF and Co-MOF/NF electrodes

The Ni-MOF/NF and Co-MOF/NF materials were prepared with the same protocol as used for CoCl_2_ or NiCl_2_·H_2_O.

### Synthesis of the BDC/NF electrode

The BDC/NF material was synthesised by following the same protocol but without CoCl_2_ and NiCl_2_·H_2_O.

### Instrumentation

The phases of the materials were analysed by Powder X-ray diffraction patterns (PXRD, X'PERT-PRO model with Cu Kα radiation sources (*λ* = 0.15406)). The morphologies of the samples were examined on a field emission scanning electron microscope (FE-SEM, FEI-quanta FEG 250). High-resolution transmission electron microscopy (HR-TEM) analyses were performed by a transmission electron microscope (JEOL/JEM 2100). X-ray photoelectron spectroscopy (XPS, k-alpha surface analysis, Thermo Fisher scientific, UK) was used to characterise the chemical composition of the electrode material. The XPS analysis was carried out at the binding energy of 0 to 1350 eV. A Fourier-transform infrared spectrometer (FT-IR, SHIMADZU, using KBr pellets) was used to find the functional groups. BET analysis was performed to analyse the surface area and porosity using N_2_ adsorption–desorption isotherms (Micromeritics Tristar 3000 instrument at 77 K).

Electrochemical measurements were carried out using an AUTOLAB PGSTAT32 electrochemical workstation in a three-electrode system in 1.0 M KOH. The NiCo-MOF/NF electrode was utilised as a working electrode, platinum rod as a counter electrode, and Ag/AgCl/KCl (3.0 M) as a reference electrode. All the measured potentials were converted into a reversible hydrogen electrode (RHE) according to eqn (5):

5 *E*_(RHE)_ = *E*_Ag/AgCl_ + 0.197 V + 0.059 pH.

Linear sweep voltammetry (LSV) and cyclic voltammetry (CV) were recorded by sweeping the potential from −0.2 to 0.8 (*vs.* Ag/AgCl) at a scan rate of 10 mV s^−1^. Electrochemical impedance spectroscopy (EIS) was recorded in the frequency range from 10^5^ Hz to 0.1 Hz. All the solutions were prepared using Millipore 18 MOhm water. All the electrochemical experiments were done under a high-purit N_2_ blanket.

### Calculations

The turnover frequency (TOF) was calculated from eqn (6):

6 TOF = *J* × *A*/4 × *F* × *m*

Here, *J* is the current density (A cm^−2^) at an overpotential of 0.27 V. ‘*A*’ and ‘*m*’ are the area of the electrode and the number of moles of the active materials on the electrode, respectively, and ‘*F*’ is the Faraday constant (96 485 C mol^−1^).

## Author contributions

Ponmuthuselvi Thangasamy and Viswanathan Subramanian contributed by designing the methodology, data analysis and writing the manuscript. All authors contributed to the data analysis, manuscript preparation and editing.

## Conflicts of interest

There are no conflicts to declare.

## Supplementary Material

NA-002-D0NA00112K-s001
