# Supplementary material for: A NiCo-MOF nanosheet array based electrocatalyst for the oxygen evolution reaction
Source: Nanoscale Adv. 2020 Apr 1;2(5):2073–9. doi: 10.1039/d0na00112k (PMC9417630; doi:10.1039/d0na00112k)
Supplement: NA-002-D0NA00112K-s001 [file NA-002-D0NA00112K-s001.pdf]

Electronic Supplementary Material (ESI) for Nanoscale Advances.
This journal is © The Royal Society of Chemistry 2020

NiCo-MOF Nanosheets Array Based Electrocatalyst for Oxygen Evolution Reaction

Ponmuthuselvi Thangasamy[a], Saravanakumar Shanmuganathan[a], Viswanathan Subramanian*[a]

[a]Department of industrial chemistry, Alagappa University, Karaikudi-630 003, Tamilnadu, India.

*Corresponding author E-mail address: rsviswa@gmail.com

SUPPORTING INFORMATION

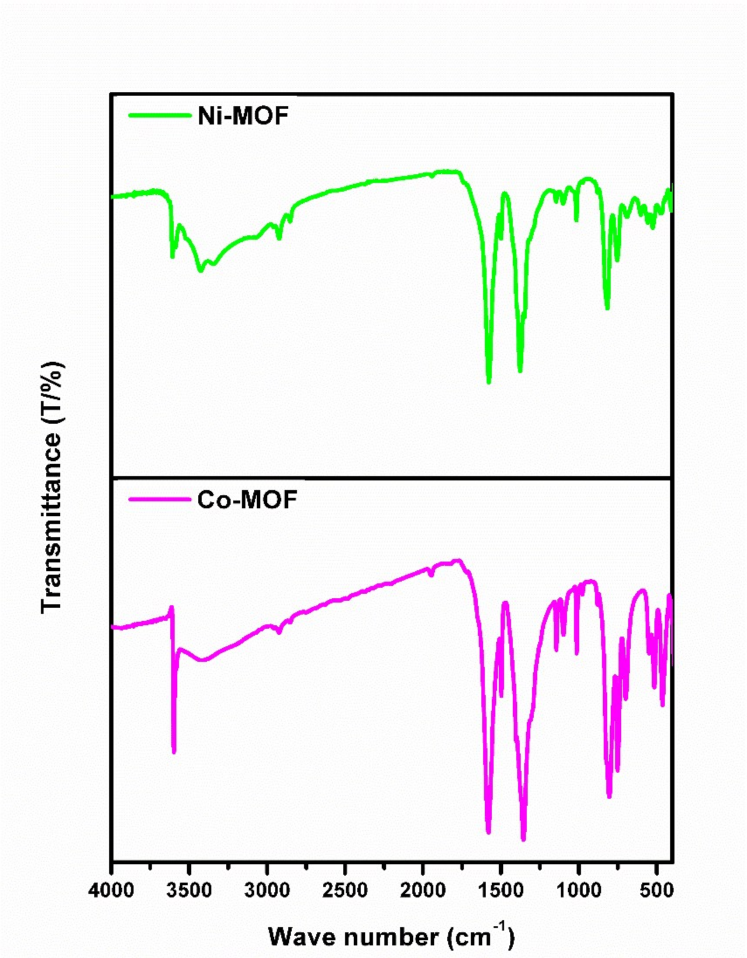

Supporting figure 1: FT-IR spectroscopy of Ni-MOF and Co-MOF

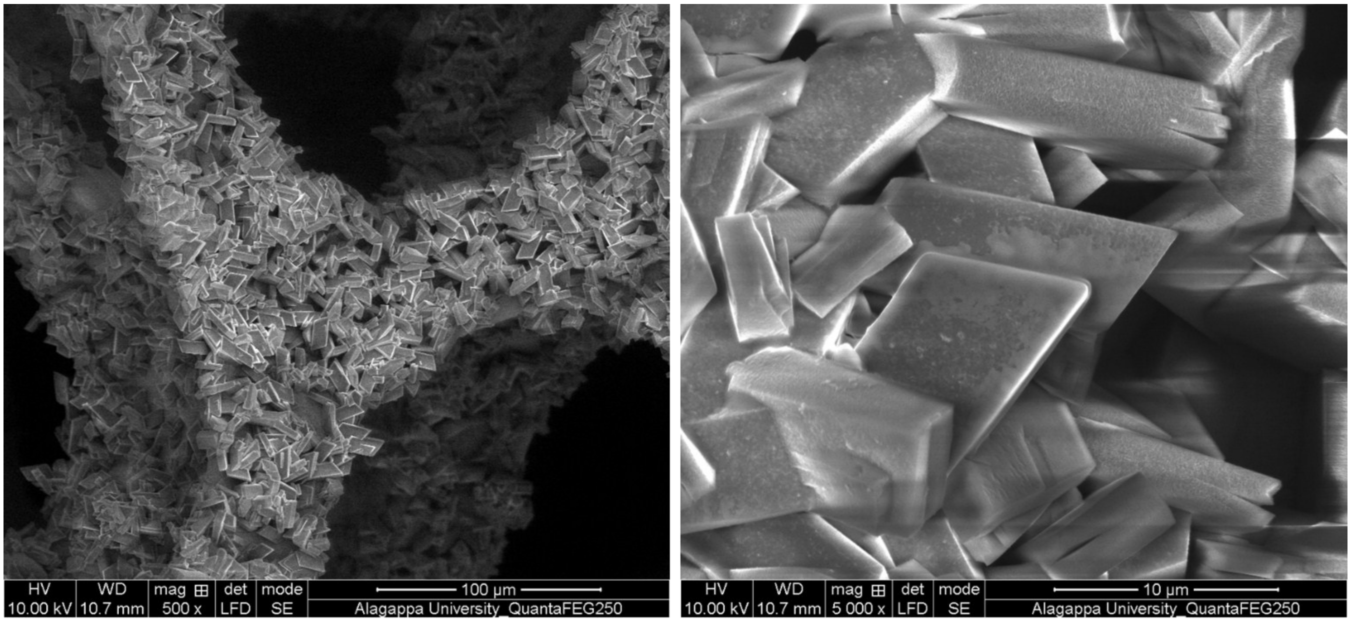

Supporting figure 2: SEM image of Ni-MOF

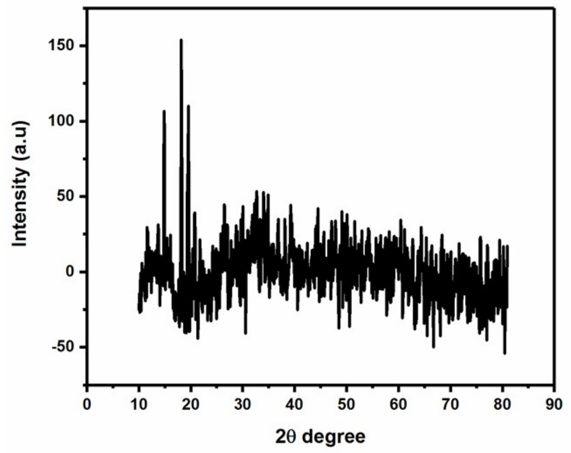

Supporting figure 3: XRD pattern of NiCo-MOF after OER measurements

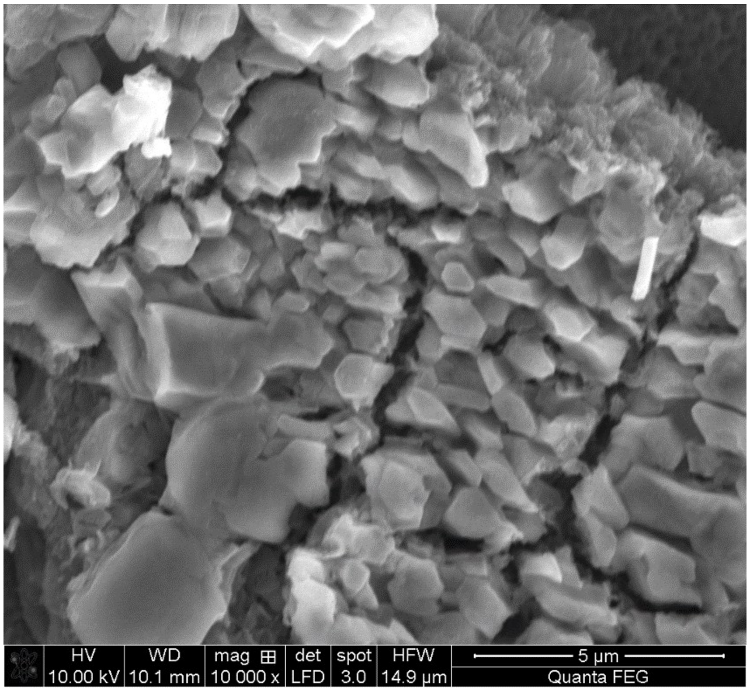

Supporting figure 4: SEM analysis of after chronoamperometric test at 1.5V for 30,000s

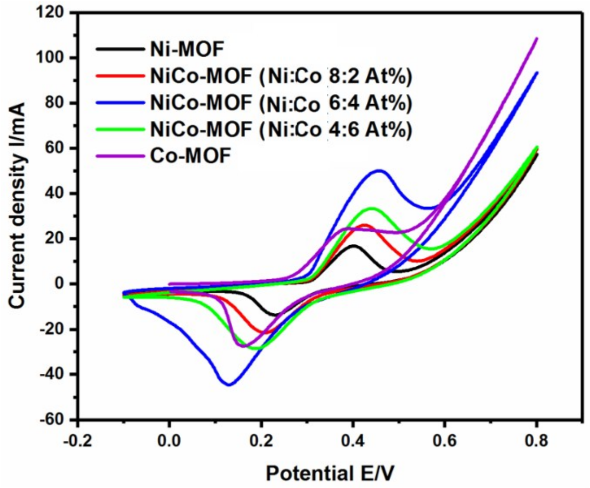

Supporting figure 5. Cyclic voltammograms of various electrode materials (Ni-MOF, NiCo-MOF (8:2 At%), NiCo-MOF (6:4 At%), NiCo-MOF (4:6 At%) and Co-MOF) scan rate at 10mV.
